# Is There an Association Between Hydration Status, Beverage Consumption Frequency, Blood Pressure, Anthropometric Characteristics, and Urinary Biomarkers in Adults?

**DOI:** 10.3390/nu17060952

**Published:** 2025-03-09

**Authors:** Joanna Frąckiewicz, Kacper Szewczyk

**Affiliations:** Department of Human Nutrition, Institute of Human Nutrition Sciences, Warsaw University of Life Sciences (WULS-SGGW), 02-787 Warsaw, Poland; kacper_szewczyk@sggw.edu.pl

**Keywords:** hydration status, dehydration, beverages, frequency consumption, dietary patterns, anthropometric measurements, urine, adults

## Abstract

Objectives: Hydration is essential for overall health; therefore, this study aimed to identify associations between hydration status and beverage consumption, anthropometric measures, and urine biochemical analyses in Polish adults. Poland was chosen due to potential regional dietary habits and hydration patterns that may influence hydration status. Methods: A total of 337 participants completed a beverage frequency questionnaire (FFQ). Blood pressure (BP), anthropometric parameters, and body composition were measured. Urine samples were analyzed for specific gravity (USG), osmolality (Uosm), and potential hydrogen value (pH). Hydration status was assessed using the WUT model (weight, urine color, thirst level), classifying participants into two groups: dehydrated (2-3 WUT components) and properly hydrated (0-1 WUT component). Odds ratios (ORs) and 95% confidence intervals (CIs) were calculated. Results: Approximately 50% of participants (n = 165) exhibited dehydration symptoms, including higher thirst levels, darker urine, and elevated USG and Uosm (*p* ≤ 0.05). Dehydrated individuals more frequently reported fatigue (*p* = 0.009), headaches (*p* = 0.024), and heavy legs (*p* = 0.002). Higher BMI (OR: 1.49), waist circumference (OR: 1.79), USG (OR: 2.29), and Uosm (OR: 1.75) increased dehydration risk. Conversely, greater consumption of tea (OR: 0.52) and non-carbonated mineral water (OR: 0.45), higher total body water (OR: 0.49), and handgrip strength (OR: 0.81) were linked to lower dehydration risk. Four dietary patterns were identified: Reasonable, Unhealthy, Minimalist, and Loving Sweet Beverages. Conclusions: Multifactorial hydration assessment, combined with preventive strategies such as regular fluid intake and weight management, may improve hydration. The WUT model and Venn diagram provide a practical tool for hydration assessment in clinical and public health.

## 1. Introduction

The term “euhydration” denotes the condition of equilibrium that exists when the amount of water taken in by the body is balanced with its loss [1]. Total body water (TBW) decreases with age, reaching approximately 75% of body weight in infants, 55% in adults, and 50% in older people [2]. In circumstances where the body experiences greater water loss than it can supply, dehydration can occur. According to the literature, mild dehydration, i.e., 1–2% loss of body weight, can impair memory and concentration, which can also result in headaches and anxiety. Weight loss of more than 2% can lead to impaired cognitive function, fatigue, and cardiovascular strain [3]. The three degrees of dehydration are as follows: mild, characterized by thirst, dry mouth, and reduced dark urine output; moderate, characterized by fatigue, headaches, and dizziness; and severe, characterized by muscle cramps, loss of consciousness, and even death [3]. This condition typically manifests in insufficient fluid intake, diarrhea, vomiting, fever, or sweating. It can also arise due to dietary indiscretion, particularly in cases of excessive salt consumption [4].

Water is a significant and essential component of the human body, performing numerous vital functions, including supporting digestive processes, contributing to thermoregulation, transporting nutrients, maintaining optimal blood pressure, and providing a moisturizing effect [1]. Maintaining adequate hydration necessitates the consumption of appropriate quantities of fluids throughout the day. This requirement is contingent on factors such as age, gender, physical activity levels, and ambient temperature. Proper hydration is crucial to health and well-being. Therefore, it is important to ensure a regular and adequate fluid intake throughout the day [5].

Because hydration plays a significant role in the body’s physiological processes, it requires precise assessment. Therefore, to ensure optimal hydration levels, it is necessary to effectively monitor its condition [3]. The assessment of hydration status can be achieved through diverse markers. The most elementary methods involve observing clinical symptoms, including thirst, skin condition, urine color, headaches, and fatigue [6]. Other methods include physiological parameters, such as weight loss, blood pressure measurement, and urine output. Laboratory methods are also employed to assess hydration status, including the measurement of urine specific gravity (USG) and osmolality (Uosm) and blood hematocrit, electrolyte, and osmolality levels [3]. A frequently employed technique for evaluating hydration is bioelectrical impedance analysis (BIA). This methodology enables the measurement of total water content (TBW), extracellular water (ECW), and intracellular water (ICW) [7]. Despite several markers to assess hydration status, there is currently no consensus among the scientific community on an unambiguous gold standard method. Combining multiple methods may improve hydration status assessment, as in the case of the Venn diagram, which combines three components, i.e., weight (W), urine color (Ucol), and thirst level (T)—WUT [8,9]. Combining different hydration markers is innovative and necessary, as single indicators often do not provide a complete result for hydration status. Therefore, using a combination of markers can minimize the risk of misinterpretation of the results [8,9].

In studies on the assessment of hydration status, authors most often use one selected method. There are no studies that use several markers of hydration status simultaneously. Therefore, this study aimed to search for associations between hydration status measured by a combination of the three methods and frequency of beverage consumption, blood pressure, anthropometric measures, hand grip strength, and urine biochemical analyses in Polish adults. Polish adults were selected for this study due to their dietary habits and the country’s distinct climatic conditions, which can significantly influence hydration status. Poland’s climate is characterized by considerable seasonal temperature variations, with cold winters and warm summers, leading to varying hydration needs throughout the year [10]. Additionally, traditional Polish diets often include a high consumption of tea and sweetened beverages, with a lower intake of plain water, which may impact overall hydration [11]. Understanding these specific factors is essential for developing targeted public health interventions to improve hydration and overall health within this population.

## 2. Materials and Methods

### 2.1. Study Design

This observational study was conducted between May 2017 and February 2020 in accordance with the Declaration of Helsinki. It was given official approval by the Ethics Committee of the Faculty of Human Nutrition and Consumer Sciences at Warsaw University of Life Sciences in Warsaw, Poland, on 11 April 2017 (Resolution No. 05p/2017). Each participant in the study gave written consent to participate in the study, which was one of the criteria for inclusion in the study.

### 2.2. Study Population and Data Collection

**Figure 1:**
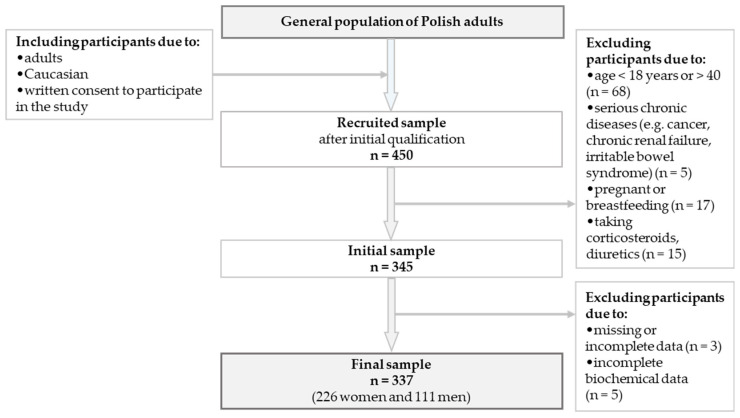
Flowchart: study design and data collection.

The convenience-sampled cross-sectional study was conducted for the general adult population. Participants were recruited from diverse social backgrounds, including urban and rural areas, varying economic statuses, and different education levels. Data were collected from 450 individuals aged 17–66, but only individuals aged 18–40 were included in further analyses to obtain a homogeneous group of respondents. Volunteers under 18 and over 40 were not included in the study because their percentage of reports was lower, and a significant part of these people most often did not meet the criteria for inclusion in the study. Analyses were conducted among 337 participants, 226 women and 111 men. The inclusion criteria encompassed individuals of Caucasian ethnicity, adults, and those capable of signing an informed consent form to participate in the study. Participants were excluded from the study if they were unable to provide informed consent or had diagnosed acute or chronic renal failure, cancer, or irritable bowel syndrome. Individuals using corticosteroids, diuretics, or other medications that could potentially affect the study outcomes were also excluded. Furthermore, participants who had experienced vomiting, diarrhea, or fever within the past three days, as well as those who were permanently bedridden or wheelchair-bound, were not eligible for inclusion. Pregnant or breastfeeding individuals, as well as those with incomplete or missing data, were also excluded. Due to the use of bioelectrical impedance analysis (BIA), individuals with endoprostheses, pacemakers, stents, or metal sutures in the heart or blood vessels were not eligible for participation. The recruitment and retention of participants throughout the study are illustrated in Figure 1.

### 2.3. Sociodemographic and Lifestyle Data

The survey method was utilized to gather general information about the respondents. The study participants completed the survey in the presence of the interviewer. The respondents were asked to provide information regarding their age (continuous variable), gender (male or female), educational level (secondary/‘I study’ or higher), place of residence (village, town, or city), self-reported economic status (very poor/poor or average/very good), self-reported health status (very poor/poor or average/very good), self-reported physical activity (no/low, moderate or high), cigarette smoking (yes or no) and body mass change in last three-month (decreased by more than 1 kg, increased by more than 1 kg, no change, or ‘I don’t know’). Furthermore, the subjects participating in the study were requested to furnish the researchers with their body weight (in kg) during the three-month period before the commencement of the study.

The respondents were asked to report the occurrence of various ailments during the day. These included fatigue (yes or no), headaches (yes or no), excessive sweating (yes or no), a feeling of fullness and an expanding abdomen (yes or no), heaviness in the legs (yes or no), swelling-related symptoms (yes or no), lower urinary tract infections (yes or no), oliguria (yes or no), very frequent urination (yes or no), and diarrhea (yes or no).

Thirst is an early indicator of dehydration [12]. Therefore, the study employed a measure of thirst. Thirst was assessed using a validated 7-point scale ranging from 1 (not at all thirsty) to 7 (very, very thirsty) [12].

### 2.4. Beverage Consumption Data and Dietary Patterns (DPs)

The dietary habits were assessed using a food frequency questionnaire (FFQ). We assessed the relative validity of the FFQ in 60 Polish participants, aged 20–30 years old from the study area, by comparing to the 4-day dietary record methods conducted twice [13]. The Spearman correlation coefficients for the associations between estimates obtained from the beverage frequency questionnaire and those from the repeated dietary record were, on average, 0.53 [13]. Subjects completed the FFQ under the supervision of an experienced dietician, thus ensuring the standardization of the procedure and the elimination of errors during data collection. Moreover, the direct interaction with the dietitian functioned as a crucial support system, addressing any ambiguities and clarifying the questionnaire’s intricacies for all respondents. A comprehensive data set was collated from the respondents, encompassing the frequency of consumption of non-alcoholic and alcoholic beverages. These beverages included: tea, coffee, herbal infusions, milk, fermented milk drinks (both natural and flavored), mineral water (both carbonated and non-carbonated), juices (fruit, vegetable, or fruit and vegetable), non-carbonated fruit drinks, sweetened carbonated drinks, tea drinks, cola drinks, energy drinks, isotonic drinks, non-alcoholic beer, beer, wine, vodka, and alcoholic drinks. Beverage consumption frequency was collected as follows—0: never; 1: <1 serving/month; 2: 1–3 servings/month; 3: 1–2 servings/week; 4: 3–4 servings/week, 5: 5 servings/week; 6: 1 serving/day; 7: ≥2 servings/day.

The DP (dietary pattern) analysis was conducted through k-means analysis, encompassing the frequency of consumption of 22 non-alcoholic and alcoholic beverages. The number of clusters in the k-means algorithm was determined arbitrarily, with four clusters selected a priori. Four dietary patterns were created: (1) Reasonable, characterized by the highest frequency of consumption of coffee, tea, herbal infusions, milk, natural and flavored fermented milk drinks, carbonated and non-carbonated mineral water; (2) Unhealthy, with the most frequently consumed beverages in this pattern including cola drinks, energy drinks, isotonic drinks, beer, wine, vodka, and alcoholic drinks; (3) Minimalist, characterized by the lowest frequency of consumption all non-alcoholic and alcoholic beverages; (4) Loving Sweet Beverages, with the most frequently consumed beverages in this pattern including juices (fruit, vegetable, or fruit and vegetable), non-carbonated fruit drinks, sweetened carbonated drinks, tea drinks, and non-alcoholic beer.

### 2.5. Blood Pressure (BP) and Anthropometric Measurements

Systolic and diastolic blood pressure measurement was conducted using a standard automatic sphygmomanometer, the SureSigns VM6 Cardiac Monitor (Philips Medical Systems, 3000 Minuteman Road, Andover, MA 01810, USA). Before the measurement, the patients were instructed to refrain from smoking or stimulant use and to have voided their bladder. This procedure was performed after a 10 min rest period. The patient was seated in a comfortable position, without a cross-legged posture, with their upper limb positioned on a table at heart level. The cuff was meticulously adjusted to accommodate the circumference of the arm and was placed on the middle third of the upper arm. Blood pressure measurements were taken in the participants in the morning, between 7:00 and 9:00 a.m. The measurement was taken in duplicate during the same visit at intervals of 1–2 min. The third measurement was taken if the difference between the first and second measurements exceeded 10 mm Hg. [14]. Standard reference values for blood pressure were defined as follows: a systolic blood pressure (SBP) measurement less than 130 mmHg and a diastolic blood pressure (DBP) measurement less than 85 mmHg, irrespective of gender [15,16].

The anthropometric measurements collected during the study encompassed several parameters, including body weight (BW), height (H), waist circumference (WC), and handgrip strength (HGS). The anthropometric measurements were conducted in duplicate by standardized protocols, with the participation of qualified researchers [17]. Participants were asked to wear minimal clothing, and the measurements were taken without shoes. BW of each participant was measured to the nearest 0.1 kg using an electronic digital scale (SECA 799, Hamburg, Germany), and H was measured using a stadiometer and recorded to the nearest 0.1 cm (SECA 220, Hamburg, Germany). The classification of BMI was conducted in accordance with the standards established by the World Health Organization (WHO) for adult subjects. BMI < 18.5 kg/m^2^ was interpreted as underweight, 18.5–24.9 kg/m^2^ as normal body weight, and BMI 25.0–29.9 kg/m^2^ as overweight and BMI ≥ 30.0 kg/m^2^ as obese [18]. The overweight and obesity groups were combined into one category. Body weight measurement before the study was recorded by the participants themselves using their personal weighing scales, but they were instructed in the proper technique for measuring body weight (in the morning, with minimal clothing, without shoes, before eating, and after using the restroom). The percentage of body weight loss (BML) for each participant was calculated based on their current body weight and body weight, taking the means from the three measurements three months ago ([BM of each day—Baseline BM]x Baseline BM^−1^ × 100). The measurement of WC was conducted utilizing a non-stretchable and tensile-resistant tape (SECA 201, Hamburg, Germany) that ensured consistent application of tension. HGS (kg) was measured to the nearest 0.5 kg at maximum effort using a hydraulic hand dynamometer (SAEHAN Corporation, Masan, Changwon, Republic of Korea). A rest period of approximately two minutes was allowed between each measurement of HGS. It was measured on the dominant and non-dominant hand. Participants assumed a standing position during the measurement. [19]. The mean values for each measurement were then calculated from the repeated readings taken [17].

### 2.6. BIA Measurements

The assessment of individual muscle mass (MM in kg and %), fat-free mass (FFM in kg and %), fat mass (FM in kg and %), total body water (TBW in kg and %), extracellular water (ECW in kg and %), and intracellular water (ICW in kg and %) was performed using a portable analyzer (Maltron BioScan 920 v1.1 software) according to the bioelectrical impedance analysis (BIA) measurement procedures. The BIA measurements were conducted in accordance with the specified conditions and the manufacturer’s protocol [20], with the subject wearing light clothing and removing all metal objects. Before BIA measurements, participants were informed about the proper preparation for analysis. Preparation for measurement included being fasted or at least 4 h after a meal, avoiding coffee at least 4 h before the measurement, avoiding alcohol 48 h before the test and physical exercise at least 12–24 h before the measurement [20]. Study participants were analyzed twice on the same day. Prior to measurement, two self-adhesive disposable skin electrodes (purchased from Maltron International Ltd., Rayleigh, United Kingdom) were applied to the appropriate site on the right hand and foot of the supine subject. The electrodes were placed in the following locations: hand—red electrode on the back of the right hand in the area of the base of the second and third fingers and black electrode on the wrist, in a straight line below the first electrode; foot—red electrode on the back of the right foot, in the area of the base of the second and third fingers, black electrode above the ankle. The device utilizes a current of 400 mA at a multi-frequency bioimpedance analysis, using 5 kHz, 50 kHz, 100 kHz, and 200 kHz. Previous studies have demonstrated that BIA-derived TBW measurements correlate with traditional hydration biomarkers and fluid balance assessments, supporting its utility in hydration research [21,22,23].

### 2.7. Biochemical Analysis in Urine

To minimize the risk of intentional overhydration before the assessment, participants were instructed not to modify their daily habits related to food and fluid intake in order to ensure a more natural and representative assessment of their usual hydration status. Moreover, prior to urine collection, they were prepared dietary-wise as for the body composition analysis. Participants took care of intimate hygiene before collecting morning urine—the first after a night’s rest. Midstream urine, 50–100 mL, was collected in a disposable urine container. Coded samples were stored at a temperature of about 4–8 degrees Celsius for up to 1 h. The samples were then stored at −20 °C until analysis for up to one month to ensure stability. The samples were assessed for color (Ucol), urine-specific gravity (USG), pH, and urine osmolality (Uosm). The analysis of the samples included the measurement of Ucol, USG, and pH. The analysis was conducted by a certified laboratory using standard methods with reference values accepted within the relevant scientific community using the refractometric method. The reference values for USG were between 1.010 and 1.030 g/cm^3^ and for pH between 5.0 and 7.5. The following values were used for Ucol: 1—straw, 2—pale yellow, 3—light yellow, 4—yellow, 5—dark yellow, 6—amber, 7—orange, 8—red, and 9—brown [24].

The Uosm of the samples was determined using a freezing point osmometer (cryoscopic method) (Marcel OS3000 osmometer, Warsaw, Poland). The osmometer was calibrated before each use to ensure accuracy. It is widely accepted that Uosm levels exceeding 700 mOsm/kg are indicative of mild dehydration, while 800 mOsm/kg are indicative of dehydration. Conversely, levels below 700 mOsm/kg are considered to reflect a normal hydration status [25].

### 2.8. Hydration Status

The hydration status of the participants was determined based on three WUT components (weight, urine color, and thirst), measured independently. The criteria for this were as follows: BML > 1%, Ucol ≥ 5, and thirst level ≥ 5, indicating dehydration [7]. If these criteria were met, 1 point was awarded; if not met, 0 points were awarded, for each component individually. The maximum dehydration score was 3, while a score of 0 indicated proper hydration. According to the above, the sum of the results obtained from the WUT components could be 0, 1, 2, or 3. In further analyses, the participants were classified based on the sum of WUT components into two groups: 0–1 WUT (proper hydration) and 2–3 WUT (dehydration).

### 2.9. Statistical Analysis

All statistical analyses were conducted using the STATISTICA 13.3 software (TIBCO Software Inc., StatSoft, Palo Alto, CA, USA) with an alpha significance level of 0.05. Additionally, the sample size for each group was initially estimated to be 67 participants (134 total) using G*Power 3.1.9.7. software. The calculations were based on the assumption of medium effects (Cohen’s d = 0.5), a statistical test for means (the Wilcoxon–Mann–Whitney test (two groups)), a statistical power of 0.8, and an alpha significance level of 0.05. The normality of distribution was examined using the Shapiro–Wilk test. The findings are articulated in the form of mean and standard deviation (SD) for continuous data or data sample proportion (%) for categorical data. The classification of participants into study groups was based on the number of WUT components, with the division into 0–1 WUT and 2–3 WUT groups. The differences between the groups were tested using the Pearson’s chi-squared test for categorical data, the Mann–Whitney U test for continuous data involving two groups. Additionally, a multivariate logistic regression model was calculated and adjusted for gender, age, and residential place to evaluate the factors corresponding with 2–3 WUT components (dehydrated status) in the study group. Odds ratios (ORs) and 95% confidence intervals (95%) were calculated. The statistical significance of the observed odds ratios was then verified using Wald’s test. Finally, for the obtained result of the factor, which was classified into 2 groups based on WUT, the effect size was obtained in the form of Cohen’s d = 1.2 (it was correctly calculated based on the difference in means and common standard deviation), and the statistical power of the test was 0.99.

## 3. Results

### 3.1. Participants Characteristic

The study involved 111 men and 226 women, with an average age of 24.1 years. The majority of respondents declared secondary education or current study (69.1%), a city as their place of residence (59.1%), average or very good self-assessment of their economic status (72.7%), and non-smoking status (88.7%). Based on the WUT components, 172 participants had 0–1 WUT component and 165 had 2–3 WUT components, which were dehydration and proper hydration. Statistically significant differences were identified between the subgroups. A significantly higher percentage of respondents with a 0–1 WUT component assessed their health as average or very good (81.4%), with high physical activity (48.8%) (Table 1). The highest percentage of respondents with a normal body weight (72.7%) and no change in body weight in the last 3 months (52.9%) was found in participants with 0–1 WUT component. In addition, respondents with 2–3 WUT components were found to have significantly higher mean T and darker Ucol compared to individuals with 0–1 WUT component.

Table 2 presents selected complaints that may be related to the hydration status of the respondents. We observed a statistically significantly higher percentage of participants with 2–3 WUT components reporting fatigue during the day (40.4%), headaches (64.2%), and heaviness in the legs (27.9%). In contrast, a statistically significantly lower percentage of respondents with 0–1 WUT components reported diarrhea (10.5%).

### 3.2. Frequency of Consumption and Symptoms of Dehydration Based on the WUT Components

The mean frequency of consuming selected non-alcoholic and alcoholic beverages, as well as DPs, according to the number of WUT components is shown in Table 3. We found statistically significant differences between the frequency of consumption of some non-alcoholic and WUT components. Subjects with 0–1 WUT component consumed tea, coffee, herbal infusions, naturally fermented beverages, still mineral water, and fruit juices significantly more often than subjects with 2–3 WUT components. We found no statistically significant differences between the frequency of consumption of alcoholic beverages and WUT components. The DPs also did not differ by group in terms of the number of WUT components.

Table 4 presents blood pressure, selected anthropometric measurements, body composition, and selected urinary biochemical analyses as a function of WUT components in the study group. We found that the respondents with 2–3 WUT components were characterized by significantly higher BMI, HC, WC, TBW (kg), and FM (kg and %) compared to the participants with 0–1 component. On the other hand, the study group with 0–1 WUT component was characterized by significantly higher HGS in the right hand and TBW (%) compared to the respondents with 2–3 WUT components. In addition, we found that the participants with 2–3 WUT components had significantly higher mean USG and Uosm compared to the respondents with 0–1 WUT component.

Anthropometric and biochemical analyses based on WUT components (Table 4) are as follows:

### 3.3. Factors Influencing Dehydration Risk Based on WUT Components

The results of the logistic regression analysis are presented in Table 5. Fatigue during the day and headache affected the dependent variable. The odds of dehydration increased by 33% (OR: 1.33; 95% CI: 1.11–1.59) in participants who experienced fatigue during the day, and the odds of dehydration increased by 46% (OR: 1.46; 95% CI: 1.15–1.78) in participants who experienced headaches. On the other hand, increasing the frequency of drinking tea and non-carbonated mineral water significantly reduced the dependent variable. Each increase in this variable reduced the probability of dehydration by 48% (OR: 0.52; 95% CI: 0.38–0.70) for tea and by 55% (OR: 0.52; 95% CI: 0.29–0.73) for non-carbonated mineral water. BMI, WC, TBW, HGS, USG and Uosm were significant in the model from the anthropometric data on body composition but also from the biochemical analyses in urine. Each 1-unit increase in BMI, WC, USG, and Uosm was linked with an increased risk of dehydration. In contrast, each 1-unit increase in HGS and TBW (%) was related to a decreased risk of dehydration. The variables gender, age, and place of residence were used to adjust the model, none of which were significant in the model.

## 4. Discussion

In the analyzed group, 11 individuals (3%) were in the 0 WUT category, 161 individuals (48%) in the 1 WUT category, 150 individuals (45%) in the 2 WUT category, and 15 individuals (4%) in the 3 WUT category. After dividing the participants into two categories—those with adequate and inadequate hydration—it was found that 51% had 0–1 WUT components, while 49% had 2–3 WUT components. This finding suggests that approximately half of the respondents exhibited signs of dehydration to varying degrees. The study established that fatigue during the day and headaches were linked with an increased likelihood of dehydration in the study group. Conversely, an increased frequency of tea and non-carbonated mineral water consumption was linked with a decreased likelihood of dehydration. Simultaneously, a 1-unit increase in BMI, WC, USG, and Uosm was linked with an increased likelihood of dehydration, while a 1-unit increase in HGS and TBW (%) was linked with a decreased likelihood of dehydration.

### 4.1. Fatigue, Headaches, and Dehydration

The present study found that fatigue during the day and headaches were factors connected with an increased likelihood of dehydration. As scientific studies indicate, even mild dehydration can cause both symptoms. The narrowing of blood vessels, precipitated by inadequate fluid intake, has been identified as a potential causative factor in the development of headaches [24,26]. Furthermore, the condition can also lead to fatigue and decreased mental and physical performance. This is attributable to a decrease in plasma volume and the resultant difficulty in delivering oxygen and nutrients to the brain and muscles. Consequently, adequate hydration is paramount for optimal cognitive and physical function, as well as overall well-being [27,28].

### 4.2. Beverage Consumption Frequency and Hydration Status

This study demonstrated a significant association between beverage consumption frequency and hydration status. Specifically, a higher frequency of tea and non-carbonated mineral water consumption was linked to a lower likelihood of dehydration. This finding was influenced by the fact that these beverages were the most frequently consumed based on frequency, with an average intake of once per day, compared to other beverages analyzed in the study group. The research indicates that water is the most frequently recommended beverage to maintain proper hydration, and it is also recommended for dietary reasons due to the lack of calories and ease of absorption [29,30]. Regular water consumption is necessary to maintain proper hydration, and the recommended daily amount of fluids to be consumed is about 2.5 L (L), which varies depending on age, gender, physical activity, and ambient temperature [5].

### 4.3. Anthropometric and Biochemical Analyses and Hydration Status

The present study demonstrated that a 1-unit increase in BMI, WC, USG and Uosm were connected with an increased likelihood of dehydration. Irrespective of the method employed to assess body hydration status, i.e., USG, Uosm, plasma osmolality, or BIA, individuals with elevated BMI were characterized by an abnormal hydration status [31,32,33,34,35]. This results indicate a potential association between hydration status and overweight/obesity, a consideration that should be taken into account within the research context. The findings of Rosinger et al. [33] provide support for the proposition that obesity is associated with water intake and hydration status, as evidenced by an analysis of the relationship between total water intake and urine osmolality in adults. Consequently, overweight/obese adults may require a greater intake of water than normal-weight adults to achieve equivalent hydration benefits [36]. This could be attributed to higher metabolic rates, increased body surface area, and greater insensible water loss in individuals with obesity, which necessitates greater fluid intake to maintain homeostasis [37]. Additionally, adipose tissue contains less water than lean mass, further contributing to differences in hydration status between individuals with varying body compositions [38]. In our study, no data were collected on food intake, but it was hypothesized that overweight/obese individuals may consume a reduced quantity of water-rich foods and beverages and be more inclined to opt for energy-dense foods, as indicated by WC and BMI [39,40]. Dietary patterns, including the consumption of processed foods high in sodium, could also play a role in hydration status, as excessive sodium intake can lead to fluid retention and altered osmoregulatory responses [41]. Moreover, physical activity levels must be considered, as overweight individuals may exhibit different sweating rates and thermoregulatory responses, potentially impacting hydration markers such as USG and Uosm [42]. Furthermore, overweight individuals may be more likely to choose sweetened beverages. In the domain of laboratory diagnostics, USG and Uosm represent two distinct yet interconnected indices that can be utilized to evaluate hydration status. The findings of this study indicate that in individuals with 2–3 WUT components, the mean USG was 1.026 g/cm^3^, while the mean urine osmolality was 767 mOsm/kg H_2_O. These findings may indicate the occurrence of dehydration to a varying degree. A statistically significant difference was observed between the two groups, with the values for the group with 2–3 WUT components being higher than those for the group with 0–1 WUT components. The elevated values for these parameters in the dehydrated group have been corroborated in other studies [34,43,44,45,46,47]. Individuals with adequate fluid intake typically produce a larger volume of urine with a lower concentration of osmotically active substances, resulting in reduced urine osmolality. Conversely, in a state of dehydration, where insufficient fluid intake occurs, the urine osmolality is elevated due to the presence of a smaller volume of highly concentrated urine. Consequently, urine osmolality serves as an indicator of the kidneys’ capacity to adequately respond to alterations in the body’s water balance [48,49]. However, it is important to acknowledge that factors such as age, sex, hormonal regulation, and renal function variability may influence urine concentration independently of hydration status, potentially acting as confounders in the assessment of dehydration risk [50]. The classification of individuals according to their hydration status is facilitated by the existence of several urine osmolality cut-off points in the literature, which are influenced by regional differences in beverage consumption and diet [1,32,43].

It was observed that an increase of 1-unit in HGS and TBW (%) was linked with a reduced likelihood of dehydration. It is acknowledged that hydration status can affect HGS, and thus it is frequently utilized as an indicator of muscle strength, as well as a measure of functional ability. Dehydration has been shown to affect muscle mass, thereby potentially leading to a decline in HGS. This is attributable to a decrease in blood volume, which in turn results in a decrease in oxygen flow to the muscles. Furthermore, dehydration has been shown to contribute to electrolyte imbalances, which can also affect HGS [27,51].

### 4.4. Strength and Limitations

The strength of our study is the participation of people from different social backgrounds, i.e., smaller and larger towns, different economic status, and different education, which allowed us to reflect the diversity of Polish society. The strength of the study was that it was conducted in direct contact, not online. This meant that any doubts respondents had when filling in the questionnaire could be clarified as they went along, thereby improving the accuracy and completeness of their responses. Another strength was that blood pressure measurements, anthropometric measurements and body composition analysis were performed by a trained person, while urine biochemical analyses were performed in a certified diagnostic laboratory. In this study, we used three methods simultaneously to assess the hydration status of the WUT—body weight loss, urine color, and thirst level, but we also used additional measurements to assess hydration, i.e., TBW, USG, and Uosm.

The present study is not without its limitations. Firstly, the sample size was not large but was consistent with the initial sample size calculations. Moreover, it is important to note that the sample in our study, while diverse, may not fully represent the general Polish population. Future studies could aim to further explore the representativeness of the sample in relation to national demographic data. Secondly, the study did not assess the participants’ dietary intake, which precluded the identification of products that could provide participants with additional water. The FFQ method was employed to collect data on beverage frequency consumption; however, this approach may have introduced errors (recall bias, underreporting) in the measurement of actual beverage consumption. Nevertheless, it should be noted that the FFQ is a well-established, validated, and widely utilized questionnaire. In addition, the WUT method (weight, urine color, thirst level) provides insight into acute rather than chronic hydration status, making it susceptible to short-term behavioral changes, such as increased fluid intake before assessment. Although participants were instructed not to modify their daily hydration habits, intentional overhydration cannot be entirely ruled out. Additionally, body mass was self-reported using personal weighing scales, which introduces potential variability in measurement accuracy and calibration. However, standardized instructions were provided to ensure consistency in weight measurements. Furthermore, thirst perception is a highly dynamic and subjective sensation influenced by environmental factors and recent fluid intake. While it may not be a reliable long-term hydration marker, its integration with other parameters enhances the overall assessment. Considering hydration is a dynamic and multifactorial process, and future studies should account for potential confounding factors, including age, sex, physical activity levels, and medical history. Additionally, future studies should consider more precise methods, such as objective biomarkers or dietary records (food and total fluid intake), to improve hydration status assessment, as these variables may influence hydration status and its attendant outcomes.

## 5. Conclusions

The findings of the present study suggest that 49% of respondents exhibited signs of dehydration (2–3 WUT components), with 51% meeting the criteria for proper hydration (0–1 WUT components), when using the three methods of assessing hydration status, namely WUT. Additionally, we incorporated other measures, such as urine specific gravity (USG) and urine osmolality (Uosm), to provide a comprehensive assessment of hydration. The results of these measurements confirmed the data obtained from the WUT components. The study group demonstrated an increased likelihood of dehydration, indicated by daytime fatigue, headaches, and elevated BMI, WC, USG, and Uosm. Conversely, a reduced probability of dehydration in the study group was observed in cases of increased frequency of tea and non-carbonated mineral water consumption and increased HGS and TBW (%). Consequently, the employment of multifaceted methods for the assessment of hydration status, the Venn diagram, in conjunction with efficacious prevention strategies encompassing regular fluid intake and body weight regulation, has the potential to enhance hydration status. The diagram helps to highlight how these factors interact and contribute to determining the overall hydration status. This approach holds promise for enhancing general health and well-being in healthy adults. Regular fluid intake and body weight regulation could be implemented in practice through targeted public health campaigns and personalized recommendations.

## Figures and Tables

**Table 1:** Study population characteristics by WUT components.

Variables	0–1 WUT (n = 172)		2–3 WUT (n = 165)		*p*–Value
	n	%	n	%	
Age (years)	24.3 ± 4.5 * 23.0 **		23.9 ± 4.5 * 23.0 **		0.565
Gender					
Male	52	30.2	59	35.8	0.281
Female	120	69.8	106	64.2	
Education					
Secondary/‘I study’	112	65.1	121	73.3	0.103
Higher	60	34.9	44	26.7	
Place of residence					
Village	32	18.6	32	19.4	0.841
Town	40	23.3	34	20.6	
City	100	58.1	99	60.0	
Economic status					
Very poor/poor	44	25.6	48	29.1	0.469
Average/very good	128	74.4	117	70.9	
Health status					
Very poor/poor	32	18.6	61	37.0	0.048
Average/very good	140	81.4	104	63.0	
Cigarette smoking					
Yes	21	12.2	17	10.4	0.594
No	151	87.8	148	89.6	
Physical activity					
No/low	47	27.3	71	43.0	0.046
Moderate	41	23.9	51	30.9	
High	84	48.8	43	26.1	
BMI					
<18.5 (kg/m^2^)	10	5.8	5	3.0	0.031
18.5–24.9 (kg/m^2^)	125	72.7	102	61.8	
≥25.0 (kg/m^2^)	37	21.5	58	35.2	
Body mass change					
Decreased by more than 1 kg	17	9.9	21	12.7	0.001
Increased by more than 1 kg	44	25.6	128	77.6	
No change	91	52.9	13	7.9	
‘I don’t know’	20	11.6	3	1.8	
Thirst level	2.77 ± 1.27 * 3.00 **		4.12 ± 1.22 * 5.0 **		0.001
Ucol	2.11 ± 0.71 * 3.0 **		4.55 ± 1.35 * 5.0 **		0.001

* mean ± SD (standard deviation); ** median; WUT: weight (W), urine color (Ucol), and thirst level (T) components; BMI, body mass index; Ucol, urine color.

**Table 2:** Selected ailments in the study group by WUT components (%).

Variables	0–1 WUT (n = 172)		2–3 WUT (n = 165)		*p*-Value
	n	%	n	%	
Fatigue during the day	36	20.7	67	40.4	0.009
Headaches	84	48.8	106	64.2	0.024
Excessive sweating	24	14.0	36	21.8	0.079
Feeling of fullness and an expanding abdomen	61	35.7	64	38.9	0.997
Heaviness in the legs	20	11.6	46	27.9	0.002
Swelling-related symptoms	45	26.1	49	30.0	0.244
Lower urinary tract infections	6	3.5	7	4.2	0.719
Oliguria	1	0.6	2	1.2	0.537
Very frequent urination	25	14.5	33	20.0	0.183
Diarrhea	18	10.5	34	20.6	0.049

WUT: weight (W), urine color (Ucol), and thirst level (T) components.

**Table 3:** Frequency of consumption of selected beverages and dietary patterns by WUT components.

Variables	0–1 WUT (n = 172)	2–3 WUT (n = 165)	*p*-Value
	Mean ± SD; Median		
Tea	5.9 ± 1.4; 6.0	4.9 ± 1.3; 5.0	0.047
Coffee	4.7 ± 1.5; 6.0	3.8 ± 1.5; 4.0	0.037
Herbal infusions	3.3 ± 0.8; 3.0	2.2 ± 0.6; 2.0	0.003
Milk	4.0 ± 1.5; 4.0	3.8 ± 1.3; 3.0	0.148
Natural fermented milk drinks	3.8 ± 1.4; 3.0	2.8 ± 1.1; 2.0	0.036
Flavored fermented milk drinks	2.1 ± 0.6; 2.0	1.9 ± 0.5; 2.0	0.938
Carbonated mineral water	2.6 ± 0.7; 1.0	2.4 ± 0.6; 2.0	0.975
Non-carbonated mineral water	6.2 ± 1.3; 7.0	5.2 ± 1.1; 6.0	0.045
Fruit juices	4.5 ± 1.3; 4.0	2.9 ± 0.4; 3.0	0.023
Vegetable juices	1.7 ± 0.3; 1.0	1.7 ± 0.4; 1.0	0.278
Fruit and vegetable juice	1.7 ± 0.5; 1.0	1.9 ± 0.6; 1.0	0.385
Non-carbonated fruit drinks	1.5 ± 0.5; 1.0	1.7 ± 0.7; 1.0	0.253
Sweetened carbonated drinks	1.8 ± 0.4; 1.0	4.9 ± 1.1; 1.0	0.415
Tea drinks	1.7 ± 0.5; 1.0	1.6 ± 0.5; 1.0	0.765
Cola drinks	1.9 ± 0.6; 2.0	2.6 ± 1.2; 2.0	0.801
Energy drinks	1.6 ± 0.5; 1.0	1.4 ± 0.3; 1.0	0.113
Isotonic drinks	1.2 ± 0.2; 1.0	1.1 ± 0.4; 1.0	0.507
Non-alcoholic beer	1.8 ± 0.4; 1.0	1.9 ± 0.5; 1.0	0.854
Beer	2.1 ± 0.4; 2.0	2.1 ± 0.3; 2.0	0.433
Wine	2.1 ± 0.6; 2.0	2.1 ± 0.4; 2.0	0.795
Vodka	1.8 ± 0.7; 1.0	1.5 ± 0.5; 1.0	0.327
Alcoholic drinks	1.7 ± 0.7; 1.0	1.5 ± 0.7; 1.0	0.395
Dietary patterns (%)			
Reasonable	30.7	22.3	0.643
Unhealthy	25.1	29.7	
Minimalist	24.7	23.8	
Loving Sweet Beverages	19.5	24.2	

WUT: weight (W), urine color (Ucol), and thirst level (T) components; SD, standard deviation.

**Table 4:** Blood pressure, anthropometric measurements, body composition, and biochemical analysis in urine by WUT components.

Variables	0–1 WUT (n = 172)	2–3 WUT (n = 165)	*p*-Value
	Mean ± SD; Median		
Blood pressure			
SBP (mmHg)	121 ± 15.54; 120	120 ± 15.21; 118.5	0.560
DBP (mmHg)	77.3 ± 8.40; 77.0	77.6 ± 8.21; 78.0	0.762
Anthropometric measurements			
BMI (kg/m^2^)	22.7 ± 3.2; 22.3	26.1 ± 3.6; 25.9	0.047
HC (cm)	96.6 ± 8.88; 95.0	99.6 ± 8.57; 99.0	0.039
WC (cm)	73.5 ± 9.51; 72.2	77.9 ± 10.68; 78.0	0.036
HGS—right hand (kg)	35.0 ± 19.3; 36.0	30.6 ±19.7; 29.8	0.032
HGS—left hand (kg)	23.1 ± 7.00; 23.0	21.6 ± 8.53; 20.0	0.105
Body composition—BIA			
MM (kg)	23.6 ± 5.09; 21.7	23.8 ± 5.56; 21.8	0.931
MM (%)	9.50 ± 2.48; 10.4	10.3 ± 3.45; 10.5	0.714
FFM (kg)	50.9 ± 10.1; 47.1	52.4 ± 11.4; 48.7	0.282
FFM (%)	76.9 ± 8.20; 77.4	76.5 ± 8.33; 76.9	0.601
TBW (kg)	37.2 ± 7.61; 34.4	40.11 ± 8.32; 37.4	0.039
TBW (%)	55.9 ± 5.76; 55.9	50.7 ± 5.83; 50.8	0.041
ECW (kg)	16.7 ± 3.64; 16.1	17.2 ± 3.77; 16.7	0.192
ECW (%)	44.7 ± 3.31; 44.4	44.9 ± 3.71; 44.7	0.391
ICW (kg)	20.8 ± 4.74; 19.0	21.0 ± 4.60; 19.5	0.278
ICW (%)	55.3 ± 3.31; 55.7	55.0 ± 3.70; 55.3	0.391
FM (kg)	14.7 ± 7.80; 13.5	19.5 ± 8.28; 19.8	0.026
FM (%)	23.1 ± 8.23; 22.5	26.4 ± 8.37; 26.6	0.038
Biochemical analysis—urine			
USG	1.014 ± 0.006; 1.017	1.026 ± 0.007; 1.024	0.001
Uosm	558 ± 166; 442	767 ± 171; 698	0.001
pH	6.13 ± 0.61; 6.00	6.10 ± 0.53; 5.90	0.375

WUT: weight (W), urine color (Ucol), and thirst level (T) components; SD, standard deviation; SBP, systolic blood pressure; DBP, diastolic blood pressure; BMI, body mass index; HC, hip circumference; WC, waist circumference; HGS, handgrip strength; MM, muscle mass; FFM, fat-free mass; TBW, total body water; ECW, extracellular water; ICW, intracellular water; FM, fat mass; USG, urine specific gravity; Uosm, urine osmolality; pH, potential of hydrogen value.

**Table 5:** Predictive model for dehydration in adults based on WUT components.

Variables	2–3 WUT				
	β ^a^	eβ ^b^	95% CI ^c^		*p*-Value ^d^
Physical activity					
No/low	1	1	1	1	
Moderate	0.051	1.07	0.84	1.32	0.625
High	0.478	1.61	0.95	2.12	0.112
Health status					
Very poor/poor	1	1	1	1	
Good/very good	0.318	1.27	0.95	1.42	0.212
Fatigue during the day	0.173	1.33	1.11	1.59	0.025
Headaches	0.457	1.46	1.15	1.78	0.031
Tea ^e^	0.421	0.52	0.38	0.70	0.009
Non-carbonated mineral water ^e^	−0.357	0.45	0.29	0.73	0.011
SBP (mmHg)	0.078	1.01	0.85	1.18	0.435
BMI (kg/m^2^)	1.116	1.49	2.53	3.60	0.036
WC (cm)	−0.413	1.79	1.26	2.42	0.021
HGS—right hand (kg)	0.367	0.81	0.68	0.97	0.047
TBW (%)	−0.717	0.49	0.32	0.76	0.004
USG	0.849	2.29	1.62	4.12	0.001
Uosm	0.555	1.75	1.27	2.48	0.002

a, estimate; b, OR-point estimate (eβ); c, 95% confidence intervals; d, the Wald test, model adjusted for gender, age and place of residence; e, the data presented relate to the frequency of consumption; WUT: weight (W), urine color (Ucol), and thirst level (T) components; SBP, systolic blood pressure; BMI, body mass index; WC, waist circumference; HGS, handgrip strength; TBW, total body water; USG, urine specific gravity; Uosm, urine osmolality.

## Data Availability

The raw data supporting the conclusions of this article will be made available by the authors on request.
